# The Diagnostic Value of Brain CT Scans in Evaluating Dizziness in the Emergency Department: A Retrospective Study

**DOI:** 10.7759/cureus.52483

**Published:** 2024-01-18

**Authors:** Abdulrahman Masood, Omar Alkhaja, Ali Alsetrawi, Fuad Alshaibani, Ahmed Awad, Zainab Habbash, Zahra Y Alyusuf, Naeema Ali, Sarah Al Mail, Tareq Al Taei

**Affiliations:** 1 Cardiology Department, Mohammed Bin Khalifa Bin Salman Cardiac Center, Riffa, BHR; 2 Radiology Department, Salmaniya Medical Complex, Manama, BHR

**Keywords:** effectiveness, single-center retrospective study, brain ct scan, dizziness vertigo imbalance, emergency

## Abstract

Background: Dizziness is a common presenting complaint to emergency departments (ED) worldwide, with causes ranging from benign to life-threatening incidents. Computerized tomography (CT) of the brain remains a common diagnostic tool used by emergency physicians; however, it appears to be of low diagnostic value, especially in patients with normal neurological assessment while carrying multiple negative implications on both the patients and the healthcare systems. Our study aims to evaluate the diagnostic value of brain CT scans in assessing patients presenting to the ED with acute dizziness.

Materials and methods: Retrospective review of medical records of patients presenting with complaints of dizziness to the ED at Salmaniya Medical Complex (SMC) who underwent a brain CT scan from January to June 2023. Collected data included patients’ demographic information, presenting complaints, and CT scan results. A multivariable analysis of factors associated with positive CT scans was performed.

Results: A total of 481 participants were enrolled in the study, representing diverse age groups as follows: 18-30 years (12.3%), 31-40 years (15.8%), 41-50 years (17.7%), 51-60 years (22.0%), and those aged over 60 years (32.2%). Among the participants, 56.3% identified as male and 43.7% as female. In terms of head trauma history, 7.1% of participants reported such incidents, while the majority (92.9%) had no history of head trauma. Exploring comorbidities, 43.5% of participants had at least one associated medical condition. Among the 481 study participants, brain CT scans revealed that the majority (93.1%) exhibited unremarkable results. The remaining cases exhibited acute events, including 5.4% with infarcts, 1.1% with hemorrhages, and 0.4% with space-occupying lesions.

Conclusion: This study provides evidence of the limited value of brain CT scans in dizzy patients with unremarkable clinical examinations. As for clinicians, it can serve as a steppingstone toward the formulation of a policy and a set of guidelines for requesting brain CT scans in patients presenting to the ED with dizziness. Future studies are suggested to provide more insights into the cost-effectiveness and utility of head CT scans in providing valuable findings.

## Introduction

Dizziness is one of the most common presenting complaints in emergency departments (ED) worldwide [1]. It is caused by various underlying pathologies ranging from benign to life-threatening conditions, including the neurological, cerebrovascular, and vestibular systems.

Detailed history taking, clinical examination, and laboratory tests of patients with dizziness can help reach a diagnosis. However, in many instances, these are complimented by computerized tomography (CT) scans of the brain to further evaluate the patients. This is especially true in cases where ischemic or hemorrhagic strokes or other serious neurological causes are suspected [2].

While brain CT scans can help reveal life-threatening causes of dizziness including cerebrovascular diseases, the overall incidence on brain CT scans of such findings in the evaluation of acute dizziness remains low [3], with only a small percentage of patients undergoing the scans found to have serious neurological conditions [4]. This is especially true as the literature suggests in cases with otherwise normal clinical neurological assessment [3].

Moreover, the sensitivity of CT scans in the early phases of ischemic strokes remains low [5]. Furthermore, the excessive and nearly routine use of CT scans in the ED may have multiple unwanted effects on both the patients and the hospitals, including unnecessary radiation exposure, increasing the patient’s length of stay in the ED [6], and further saturating the healthcare system while applying additional potentially avoidable costs.

Between the questionable benefits of using CT scans in patients presenting with dizziness at the ED and the associated negative implications, our study evaluates the diagnostic value of Brain CT scans in assessing patients with dizziness in the ED in a retrospective single-center sample.

## Materials and methods

Study design and setting

A retrospective cross-sectional review of medical records of patients presenting with the complaint of dizziness to the ED at Salmaniya Medical Complex (SMC) who underwent a brain CT scan from January 2023 to June 2023 was done. Collected data included patients’ demographic data, presenting complaints, and brain CT scan results.

Study population

This study included all patients who presented to the ED aged >18 years old with dizziness, Glasgow Coma Scale (GCS) of 15, had no neurological deficit on neurological examination, and had a brain CT scan done. Patients who had positive neurological deficits, alcohol intoxication, pregnancy, peripheral causes of dizziness, hypotension, and hypoglycemia were all excluded from the study.

Ethical consideration

This study was ethically approved by the governmental hospital's ethical committee, Ministry of Health, Kingdom of Bahrain (Research approval serial no: 50230533). The ethical principles of the Helsinki Declaration were followed in this study.

Statistical analysis

The collected data were compiled using Microsoft Excel. Before analysis, a rigorous data quality assurance process was implemented, encompassing comprehensive checks for accuracy and consistency. Entries containing information about patients under 18 years of age were meticulously identified and subsequently removed from the dataset, aligning with the study's emphasis on adult populations. To summarize the demographic and health-related characteristics of the study participants, descriptive statistics were employed. Categorical variables, including age groups, gender, head trauma history, and comorbid conditions, were presented through frequencies and percentages. The associations between clinical variables and positive CT scan findings were explored using chi-square tests and Fischer exact tests.

Furthermore, a multivariable logistic regression analysis was conducted using SPSS version 27 (IBM Corp., Armonk, NY). This analysis aimed to identify factors independently associated with positive CT scan results. Odds ratios (ORs) alongside their corresponding 95% confidence intervals (CI) were calculated to quantitatively assess the strength of associations. All statistical tests were conducted as two-tailed tests, and a significance level of p < 0.05 was adapted to establish statistical significance.

## Results

Demographic and health characteristics

A total of 481 participants were enrolled in the study, representing a diverse age group as follows: 18-30 years (12.3%), 31-40 years (15.8%), 41-50 years (17.7%), 51-60 years (22.0%), and those aged over 60 years (32.2%). Among the participants, 56.3% were males, and 43.7% were females. In terms of head trauma history, 7.1% reported such incidents, while 92.9% had no history of head trauma. Exploring comorbidities, 43.5% of participants had at least one additional medical condition. The most prevalent comorbidities were hypertension (31.4%) and diabetes mellitus (20.2%). Less common conditions included dyslipidemia (3.3%), cerebrovascular accident (2.7%), ischemic heart disease (2.3%), malignancy (1.9%), hypothyroidism (1.5%), sickle cell disease (1.2%), chronic kidney disease (0.8%), asthma (0.8%), acquired immune deficiency syndrome (0.6%), and connective tissue disease (0.4%) (Table 1).

**Table 1:** Demographic and health characteristics of study participants (n=481).

Variable		Frequency	%
Age Group (years)	18–30	59	12.3
	31–40	76	15.8
	41–50	85	17.7
	51–60	106	22.0
	>60	155	32.2
Gender	Male	271	56.3
	Female	210	43.7
Head Trauma	No	447	92.9
	Yes	34	7.1
Comorbidities	Any Comorbid Condition	209	43.5
	Hypertension	151	31.4
	Diabetes Mellitus	97	20.2
	Dyslipidemia	16	3.3
	Cerebrovascular Accident	13	2.7
	Ischemic Heart Disease	11	2.3
	Malignancy	9	1.9
	Hypothyroidism	7	1.5
	Sickle Cell Disease	6	1.2
	Chronic Kidney Disease	4	0.8
	Asthma	4	0.8
	Acquired Immune Deficiency Syndrome	3	0.6
	Connective Tissue Disease	2	0.4

Brain CT scan findings

Among 481 study participants, brain CT scans revealed that the majority (93.1%) had unremarkable results. The remaining cases exhibited acute events, including 5.4% with infarcts, 1.1% with hemorrhages, and 0.4% with space-occupying lesions (Table 2).

**Table 2:** Brain CT scan findings of study participants (n=481).

Brain CT Scan Results		Frequency	%
Unremarkable		448	93.1
Acute Event	Infarct	26	5.4
	Hemorrhage	5	1.1
	Space Occupying Lesion	2	0.4

Associations between clinical variables and positive brain CT scan findings

Exploring the relationship between clinical variables and positive CT scan findings, a significant association was observed between age groups and positive CT scans (p<0.01). Notably, the highest percentage of positive CT scans was seen in the 41-50 age group (15.3%). Additionally, a significant association was noted for the presence of any comorbid condition (p=0.02), indicating a positive CT scan rate of 10% among participants with comorbidities, compared to 4.4% in those without comorbidities. However, no specific comorbid condition exhibited a significant association with positive CT scan findings (p>0.05). Moreover, no statistically significant associations were identified between positive CT scan findings, gender, history of head trauma, or the frequency of reported dizziness (Table 3).

**Table 3:** Association between clinical variables with positive brain CT scan findings

Variable		Positive CT scan	%	P-value
Age Group (years)	18–30	2	3.4	<0.01
	31–40	3	3.9	
	41–50	13	15.3	
	51–60	2	1.9	
	>60	13	8.4	
Gender	Male	19	7.0	0.88
	Female	14	6.7	
Head Trauma	No	31	6.9	0.82
	Yes	2	5.9	
Comorbidities	Any Comorbid Condition	21	10.0	0.02
	Hypertension	15	9.9	0.08
	Diabetes Mellitus	7	7.2	0.88
	Dyslipidemia	2	12.5	0.30
	Ischemic Heart Disease	2	18.2	0.17
	Hypothyroidism	1	14.3	0.39
	Chronic Kidney Disease	0	0.0	1.00
	Cerebrovascular Accident	0	0.0	1.00
	Sickle Cell Disease	0	0.0	1.00
	Malignancy	0	0.0	1.00
	Asthma	0	0.0	1.00
	Connective Tissue Disease	0	0.0	1.00
	Acquired Immune Deficiency Syndrome	0	0.0	1.00
Frequency of Dizziness	Single Episode	27	6.6	0.62
	Multiple Episodes	6	8.2	

Multivariable analysis of factors associated with positive brain CT scans

In the multivariable analysis aimed at identifying factors linked to positive CT scan findings, the presence of at least one comorbid condition emerged as the sole significantly associated independent factor (OR = 2.33, p = 0.04). Conversely, age groups and gender did not demonstrate statistically significant associations with positive CT scan findings (Table 4).

**Table 4:** Multivariable analysis of factors associated with positive brain CT scans

Variable		OR	95% CI	P-value
Age Group (years)	18–30	Reference Group		
	31–40	1.04	0.17–6.47	0.97
	41–50	3.95	0.83–18.74	0.08
	51–60	0.39	0.05–2.94	0.36
	>60	1.62	0.33–7.98	0.55
Gender	Male	Reference Group		
	Female	1.1	0.52–2.29	0.82
Comorbidities	No Comorbidities	Reference Group		
	≥1 Comorbid Condition	2.33	1.04–5.20	0.04

## Discussion

Our study explored the effectiveness of brain CT imaging in the evaluation of patients presenting to the ED with dizziness without neurological deficits. Our findings showed that there was little benefit of the routine use of CT brain in the acute setting for dizziness evaluation.

Dizziness is one of the most common symptoms for presenting to the ED. It is a challenging problem for clinicians as it can be a manifestation of multiple disorders, ranging from serious life-threatening diseases to a normal human body response [7]. Dizziness can be central and peripheral. Central causes include cerebrovascular disease, vestibular migraine, and meningiomas. Peripheral causes include benign paroxysmal positional vertigo, Meniere’s disease, and otosclerosis. Other causes include psychiatric illnesses, cardiovascular diseases, orthostatic, and drug-induced [8]. There is no specific investigation to diagnose the underlying cause of dizziness, thus diagnosis depends primarily on clinical factors. Focused history-taking with special attention to onset, duration, the evolution of symptoms, triggers to these episodes, and other associated symptoms, in addition to physical examination, including neurological, cardiac, and HEENT assessment [7,8]. CT scan has been extensively used in dizzy patients presenting to the ED, despite it being time-consuming, costly, and having radiation exposure. This study goes hand in hand with the literature, where brain CT scan has a very low diagnostic value in patients presenting to ED with dizziness.

Alawneh et al. and Staibano et al. both noted a significantly higher percentage of females presenting to the ED with dizziness [9,10]. However, in our study, males (56.3%) were more than females (43.3%), which goes hand in hand with Mitsunaga et al. findings of male predominance in the same setting of patients [11]. Despite that, the majority of patients included in this study were elderly patients more than 60 years of age (32.2%), and the highest positivity rate for the brain CT scans was noted in patients aged 41 to 50 (15.3%). Similarly, Alawneh et al. observed that most patients were older than 44 years of age [9]. In addition, Mitsunaga et al. found that the mean age of patients presenting to ED with dizziness and having CT brain done was 64 years of age [11]. Other studies had the same demographics as most of the patients were elderly and of a female gender [12,13].

According to the findings of this study, in 481 patients presenting to the ED with dizziness, 448 (93.1%) had unremarkable findings in brain CT scans. Our findings are comparable to previous studies that assessed the efficacy of routine brain imaging for patients with dizziness and concluded that it is of limited diagnostic value, time-consuming, and not as cost-effective [9,10,14,15]. As previously studied, CNS causes of dizziness are uncommon, comprising only 4%-6% of ED visits for dizziness [16]. Disorders presenting with dizziness that are not life-threatening typically do not require a brain CT scan and are frequently diagnosed clinically [16].

Having any comorbidity is significantly associated with a positive CT brain (p=0.02), with a rate of 10% in those with comorbidities and 4.4% for those without a comorbid condition. The most prevalent comorbidities were hypertension (31.4%) and type II diabetes mellitus (20.2%), while the least prevalent were AIDs (0.6%) and connective tissue disorders (0.4%). Alawneh et al. concluded that both type II diabetes mellitus and hypertension are the most common comorbidities in patients presenting with dizziness [9]. Since psychological evaluation requires a thorough clinical examination that may not be applicable in the emergency setting, this study did not investigate any underlying psychological illnesses, such as anxiety or depression, that might be underlying the patient’s presentation. However, Staibano et al. investigated the possibility of these illnesses [10], and it was suggested that psychological intervention can improve symptoms, decrease disability, and reduce impairment [10,17,18].

This study provides clear evidence of the limited value of routine CT brain scans in dizzy patients coming to ED without any neurological deficits. For clinicians, this study can serve as a steppingstone toward the formulation of a policy and a set of guidelines for requesting brain CT scans. This study was limited by its retrospective design. It may have resulted in the lack of some patient data or the unavailability of accurate medical records. In addition, some patients have difficulty distinguishing between dizziness and vertigo, which subjects it to the clinician's judgment in labeling the patient with vertigo or dizziness and determining whether a brain CT scan is required. Even though imaging in ED patients with dizziness is a well-studied topic, this is a single-center study with a local approach; therefore, the findings may not apply to other institutions. Additional research is required to apply these results to other secondary care hospitals.

## Conclusions

In conclusion, our study showed that routine use of brain CT scans in patients with dizziness without neurological deficits has low diagnostic value and is not as cost-effective. Guidelines regarding ordering CT brain are crucial to ensure accurate patient diagnosis and avoid patient delay in the ED. As for clinicians, this study can serve as a stepping stone toward the formulation of a policy and a set of guidelines for requesting CT brain scans. Future studies are suggested to provide more insights into the cost-effectiveness and utility of brain CT scans in providing valuable findings. 
